# Asia-Pacific venous thromboembolism consensus in knee and hip arthroplasty and hip fracture surgery: Part 1. Diagnosis and risk factors

**DOI:** 10.1186/s43019-021-00099-y

**Published:** 2021-06-19

**Authors:** Srihatach Ngarmukos, Kang-Il Kim, Siwadol Wongsak, Thanainit Chotanaphuti, Yutaka Inaba, Cheng-Fong Chen, David Liu, Asia-Pacific (AP) Region Venous Thromboembolism (VTE) Consensus Group, Aasis Unnanuntana, Alvin Tan, Anthony Pohl, Apisak Angsugomutkul, Apisit Patamarat, Arak Limtrakul, Aree Tanavalee, Azhar Merican, Azlina Abbas, Badrul Shah Badaruddin, Boonchana Pongcharoen, Bui Hong Thien Khanh, Cao Li, Chaithavat Ngarmukos, Charlee Sumettavanich, Chavanont Sumanasrethakul, Chavarin Amarase, Chee-Ken Chan, Chong Bum Chang, Chotetawan Tanavalee, Christopher Scott Mow, Chumroonkiet Leelasestaporn, Chun Hoi Yan, Dang-Khoa Tran, David Campbell, Edi Mustamsir, Edsel Fernandez Arandia, Eun Kyoo Song, G Ruslan Nazaruddin Simanjuntak, Hirotsugu Muratsu, Hyonmin Choe, Jamal Azmi Mohammad, Jason Chi Ho Fan, Ji Hoon Bae, Ji-Wan Kim, Jose Antonio San Juan, Jose Fernando C Syquia, Jun-Ho Kim, KiKi Novito, Kriskamol Sithitool, Manoon Sakdinakiattikoon, Masaaki Matsubara, Mel S Lee, Mohamad Zaim Chilmi, Myint Thaung, Myung Chul Lee, Narathorn Kongsakpaisal, Ngai Nung Lo, Nicolaas Budhiparama, Nikom Noree, Nobuhiko Sugano, Paphon Sa-ngasoongsong, Pariwat Taweekitikul, Peter Bernardo, Piti Rattanaprichavej, Piya Pinsornsak, Po-Kuei Wu, Pongsak Yuktanandana, Pruk Chaiyakit, Rahat Jarayabhand, Rami Maher Sorial, Ross W Crawford, Ryuji Nagamine, Saradej Khuangsirikul, Saran Tantavisut, Satit Thiengwittayaporn, Seng Jin Yeo, Sukit Saengnipanthkul, Supparurk Suksumran, Surapoj Meknavin, Thakrit Chompoosang, Than Win, Thana Narinsorasak, Thana Turajane, Thanarat Reancharoen, Tokifumi Majima, Ukrit Chaweewannakorn, Viroj Kawinwonggowit, Viroj Larbpaiboonpong, Wanshou Guo, Weerachai Kosuwon, Wei Chai, William J. Maloney, Yee Hong Teo, Yixin Zhou, Yunsu Chen, Yutthana Khanasuk

**Affiliations:** 1 Department of Orthopaedics, King Chulalongkorn Memorial Hospital and Faculty of Medicine, Chulalongkorn University, Bangkok, Thailand; 2 Department of Orthopaedic Surgery, Center for Joint Diseases, Kyung Hee University Hospital at Gangdong, 892 Dongnam-ro, Gangdong-gu Seoul, Seoul, 134-727 Korea; 3 Department of Orthopaedic Surgery, School of Medicine, Kyung Hee University, Seoul, Korea; 4 Department of Orthopaedics, Faculty of Medicine Ramathibodi Hospital, Mahidol University, Bangkok, Thailand; 5 Department of Orthopaedics, Phramongkutklao Hospital and College of Medicine, Bangkok, Thailand; 6 Department of Orthopaedic Surgery, Yokohama City University Hospital, 3-9 Fukuura, Kanazawa-ku, Yokohama, Japan; 7 Department of Orthopaedics and Traumatology, Taipei Veterans General Hospital, Taipei, Taiwan; 8 Department of Orthopaedics, School of Medicine, National Yang-Ming University, Taipei, Taiwan; 9 Gold Coast Centre for Bone & Joint Surgery, Gold Coast, Australia

## Background

Postoperative venous thromboembolism (VTE) is a significant cause of morbidity and mortality in patients undergoing knee and hip arthroplasty and hip fracture surgery; however, VTE is considered potentially preventable with several modalities of prophylactic management [1, 2]. The VTE prevention guidelines by the American Academy of Orthopaedic Surgeons (AAOS) or the American College of Chest Physicians (ACCP) have been implemented in most countries in Asia [3–5]. However, according to these guidelines, there are some concerning issues and complications related to VTE prophylaxis in major joint replacement and hip fracture surgeries because of differences in the healthcare systems and cultural aspects [6, 7].

Among orthopedic surgeons who practice in the Asia-Pacific (AP) Region, some alternative options for VTE prevention in knee and hip arthroplasty and hip fracture surgery are somewhat necessary. These Asian-specific guideline/consensus statements are expected to provide better patient outcomes and compliances. Therefore, in a one-year period, we have obtained an AP consensus agreement on VTE prophylaxis in knee and hip arthroplasty and hip fracture surgery in Asian patients among AP orthopedic experts. According to the results of the consensus statements, some accepted methods of VTE prophylaxis are different from those published in international guidelines regarding the details of diagnosis and risk factors and methods of prophylaxis.

We hope that this AP VTE consensus will provide orthopedic surgeons who practice in the AP Region appropriate options for VTE diagnosis and prevention methods that benefit their patients with fewer complications.

## Methods

**Fig. 1:**
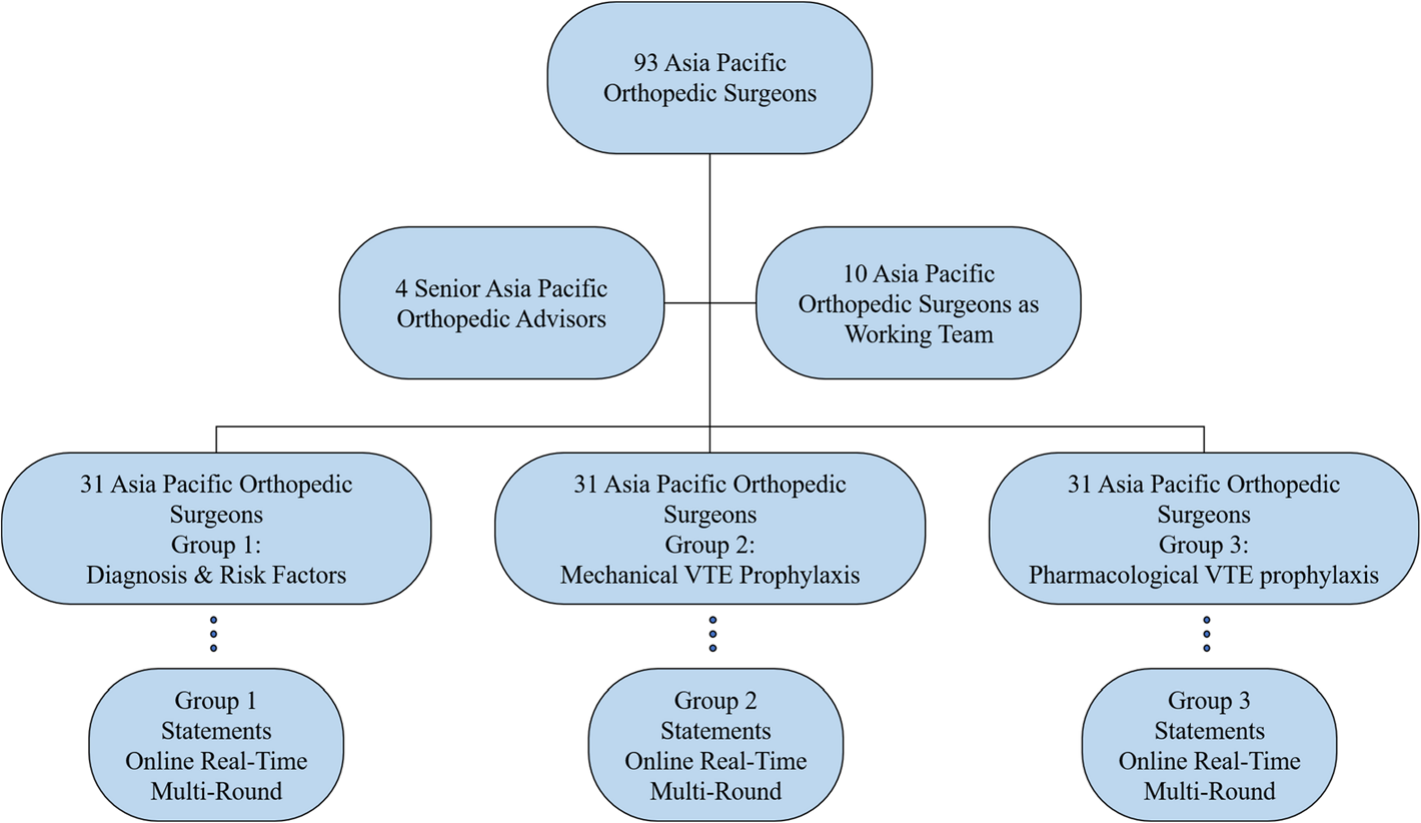
Demonstration of method and procedures for formation of AP venous thromboembolism consensus

The consensus was instructed using the modified Delphi method for agreement [8, 9]. The working team used a real-time Delphi multiround online software (https://calibrum.com), responding anonymously [10]. After receiving agreement of 93 AP orthopedic experts to join the group, all experts were divided into three groups, including group 1: diagnosis and risk factors; group 2: mechanical VTE prophylaxis; and group 3: pharmacological VTE prophylaxis (Fig. 1).

The first-round real-time online survey was begun on 18 October 2019. There were four rounds of the survey and an additional round for consensus voting. The consensus was successfully finished on 11 September 2020. In each round, experts could repeatedly view and comment on documents via the Internet until the end of the survey time. Based on evidence-based medicine and experts opinions, the five-round survey has optimized AP experts’ agreement on debatable issues in VTE prophylaxis in knee and hip arthroplasty and hip fracture surgery before consensus voting, in terms of statements, recommendations, justifications, and references. Given the approximately one-month period of each round, all experts could have several opportunities to access the survey for the online response. All statements in the survey were accompanied by a text box allowing comments in qualitative responses to be made anonymously. Besides open comments, experts were requested to rate their agreement on each statement according to a Likert scale of 1–9, (1 = strongly disagree, 9 = strongly agree) [11].

**Fig. 2:**
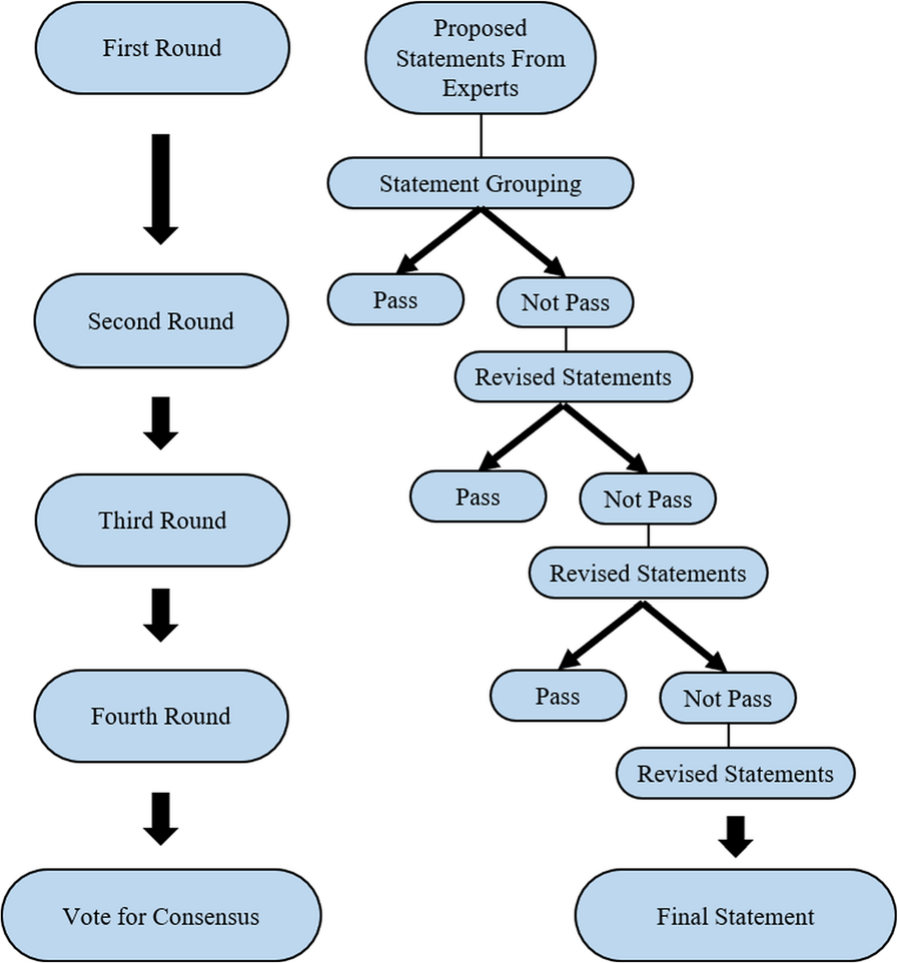
Algorithm demonstrating four-round consensus survey using modified Delphi method and the final voting for consensus

After closing of the survey in each round, the working team and assistants read the responses for all statements, including comments or suggestions, and also searched out more evidence-based medicine data-related commented issues. For agreement evaluation, all statements were assessed into three categories: (1) Agreement: Likert 7, 8, and 9; (2) Neutral: Likert 4, 5, and 6; and (3) Disagreement: Likert 1, 2, and 3. All passed statements needed to have ≥75% agreement from voters [9]. Any statements having < 75% agreement were to be reviewed, revised, and resubmitted in the next round of the survey. Again, all AP experts were asked for agreements and comments until the final round (Fig. 2) [9]. After four rounds of surveys, all passed statements were finally sent for online voting [9]. The criteria of voting for consensus were as follows: (1) Unanimous consensus: 100% agreement; (2) Strong consensus: ≥ 75% agreement; (3) Weak consensus: > 50%, < 75% agreement [9].

## Statements of Group 1: Diagnosis and Risk Factors

### 1. Which surgical procedures are considered knee arthroplasty, hip arthroplasty, and hip fracture surgery?

#### Recommendation

Knee arthroplasty includes total knee arthroplasty (TKA), unicompartmental knee arthroplasty (UKA), and patellofemoral arthroplasty (PFA). Hip arthroplasty includes total hip arthroplasty (THA), hip hemiarthroplasty, and hip resurfacing arthroplasty. Hip fracture surgery includes both internal fixation and hip arthroplasty for treatment of hip fracture in adults, including femoral neck fracture, intertrochanteric fracture, and subtrochanteric fracture.

##### Delegate vote

Agree 100%, Disagree 0%, Abstain 0% (Unanimous Consensus).

#### Justification

TKA involves surgical removal of damaged bone and cartilage of the distal femur and proximal tibia, followed by replacement with a corresponding metallic femoral component and tibial component with a polyethylene insert [12]. UKA involves surgical removal of damaged bone and cartilage of only the medial compartment or lateral compartment of the tibiofemoral joint, and then replacement with a corresponding metallic femoral component and a tibial component with a polyethylene insert [13]. PFA involves surgical removal of damaged bone and cartilage from the patella’s underside and the trochlear groove of the femur, followed by replacement with metal and plastic components [14].

THA generally uses an acetabular component with a bearing surface, a femoral component, and a femoral head prosthesis [15, 16]. Hip hemiarthroplasty involves surgical removal of the femoral head and part of the femoral neck, followed by replacement with a femoral prosthesis [17, 18]. No procedure is performed on the acetabulum [17, 18]. Total resurfacing arthroplasty involves partial removal of the femoral head and then capping it with a similar-sized spherical metallic prosthesis [19]. The acetabulum is replaced with a monobloc metal component [19].

Internal fixation of the hip fracture uses devices to stabilize the fracture, including multiple screws with various designs, sliding screw-plates, cephalomedullary nails, and other devices of similar function [20].

### 2. Does UKA have a similar risk of postoperative VTE when compared to TKA?

#### Recommendation

No, UKA has a lower risk of postoperative VTE than TKA.

Delegate vote: Agree 97.3%, Disagree 2.7%, Abstain 0% (Strong Consensus).

#### Justification

Several studies have demonstrated a lower incidence of postoperative VTE in UKA when compared to TKA [21–24]. A recent systemic review and meta-analysis regarding the incidence of VTE after knee arthroplasty, including 33,232 UKAs and 229,166 TKAs, demonstrated that UKA has a significantly lower risk of postoperative VTE than TKA [22]. Remaining data analyzed from randomized controlled trials (RCTs) and extensive cohort studies show statistically nonsignificant trends toward lower risk of VTE in UKA [22]. Another systemic study comparing outcomes of UKA and TKA reported a lower incidence of VTE after UKA (1.75%) when compared to TKA (4.1%) [21]. However, these results do not reach statistical significance [21]. One study was a matched-pair prospective study from Singapore, which can represent the Asian population [23]. Another prospective study in 3349 patients undergoing knee replacement found an incidence of postoperative deep vein thrombosis (DVT) of 0.3% in the UKA patients vs. 2.2% in the TKA patients, with a significant difference [24].

### 3. Does hemi-hip arthroplasty have a similar risk of postoperative VTE compared to THA in hip fracture patients?

#### Recommendation

Yes, currently available evidences demonstrate that hemiarthroplasty has a similar risk of postoperative VTE compared to THA in hip fracture patients.

Delegate vote: Agree 97.3%, Disagree 2.7%, Abstain 0% (Strong Consensus).

#### Justification

There was no significant difference in VTE incidence between hip fracture patients who underwent a THA and those who underwent a hemiarthroplasty [25, 26]. Data from The National Surgical Quality Improvement Program Database show that the rate of DVT requiring therapy in hemiarthroplasty and THA in patients with femoral neck fracture was similar at 0.8% [25]. The rates of pulmonary embolism (PE) in hemiarthroplasty and THA in femoral neck fracture patients were 0.6% and 1.0%, respectively [20]. In a propensity score-matched, population-based study of patients with femoral neck fracture, the rate of DVT was 0.9% in the hemiarthroplasty group compared to 1.1% in the THA group [26]. Another study showed that the rate of PE was 1.2% in the hemiarthroplasty group compared to 0.9% in the THA group [19]. Additionally, patients undergoing hip arthroplasty for fracture treatment are at significantly higher risk of VTE than patients who have undergone an elective THA [27].

### 4. Is the incidence of VTE after knee and hip arthroplasty or hip fracture surgery lower in patients of Asian than Caucasian ethnicity?

#### Recommendation

Yes, the incidence of VTE after knee and hip arthroplasty or hip fracture surgery is lower in Asian than in Caucasian ethnicities.

Delegate vote: Agree 94.6%, Disagree 0%, Abstain 5.4% (Strong Consensus).

#### Justification

The incidence of postoperative VTE is likely lower in people of Asian ethnicity than in those of Caucasian ethnicity, after knee and hip arthroplasty or hip fracture surgery [28–32]. A cohort study shows that eight of 3070 (0.26%) Asian patients had VTE after THA, and 585 of 57,559 (1%) Caucasian patients had VTE after THA [30]. Moreover, the Asian patients had a significantly lower likelihood of 90-day VTE when compared with Caucasian patients [30]. A Singaporean cohort study of Asian patients who underwent TKA from 2006 to 2014 showed that eight of 966 patients (0.82%) had VTE after TKA [28]. Another cohort study of consecutive Asian patients undergoing lower limb major orthopedic surgery showed that the those who underwent TKA, THA, and hip fracture surgery had symptomatic VTE rates of 1.4%, 1.0%, and 0.6%, respectively [29]. A prospective study of 1608 Asian patients who underwent TKA and THA showed that the incidences of VTE after TKA and THA were 4.31% and 0.88% [31]. A large epidemiologic study from the Korean national database, which investigated the incidence of VTE after major surgery in Asia, demonstrated the rate of VTE after TKA, THA, and hip fracture surgery to be 1.08%, 0.98%, and 1.60% [32].The rate of DVT after TKA, THA, and hip fracture surgery was 0.71%, 0.62%, and 0.66% [32]. Other results showed that the rate of PE after TKA, THA, and hip fracture surgery was 0.37%, 0.36%, and 0.94% [32]. This study concluded that the rates of postoperative VTE are lower in Korean than in Caucasian populations [32]. Another prospective study of TKA in 227 Korean patients, performed by a single surgeon, reported the rate of proximal DVT to be 3.03% and that of distal DVT to be 23.57%, with no symptomatic PE, which confirmed the low VTE incidence in the Koreans undergoing TKA [33].

The Seventh ACCP Conference on Antithrombotic and Thrombolytic Therapy in 2004 documented the rate of total DVT in Caucasian patients after TKA, THA, and hip fracture surgery as 41–85%, 42–57%, and 46–60% and the rate of proximal DVT in Caucasian patients after TKA, THA, and hip fracture surgery as 5–22%, 18–36%, and 23–30% [34]. This study also showed that the rate of PE in Caucasian patients after TKA, THA, and hip fracture surgery was 1.5–10%, 0.9–28%, and 3–11% [34].

A systematic review and meta-analysis of an Asian population showed that the rate of DVT in patients who underwent TKA was 42.5%, and the rate of DVT in patients who underwent THA or hip fracture surgery was 25.8% [7]. Furthermore, the rate of PE in patients who underwent TKA was 0.5%, and the rate of PE in patients who underwent THA or hip fracture surgery was 0.3%. The study concluded that the pooled rates of proximal and symptomatic DVT in Asians were lower than those of the Caucasian reports [7]. Another meta-analysis that studied Asian patients who underwent TKA showed that the incidence of symptomatic PE was 0.01%. The incidences of overall DVT, proximal DVT, and symptomatic DVT were 40.4%, 5.8%, and 1.9% [35].

### 5. Are there different degrees of VTE risk in Asian patients undergoing knee and hip arthroplasty or hip fracture surgery?

#### Recommendation

Yes, Asian patients undergoing knee and hip arthroplasty or hip fracture surgery have standard and elevated VTE risks.

Delegate vote: Agree 94.6%, Disagree 0%, Abstain 5.4% (Strong Consensus).

#### Justification

The incidence of postoperative VTE in patients of Asian ethnicity is likely lower than in those of Caucasian ethnicity after knee and hip arthroplasty or hip fracture surgery [7, 28, 35]. However, there are no specific studies regarding the VTE risk in Asian patients undergoing knee and hip arthroplasty or hip fracture surgery. Asian patients should be classified as having standard VTE risk and elevated VTE risk.

Patients at standard VTE risk have no histories or medical conditions associated with VTE before surgery. Patients at elevated VTE risk already have histories or medical conditions associated with VTE before surgery. The exception to these statements involves certain surgical factors that elevate VTE risk from the usual, such as bilateral procedures and prolonged surgical time (> 2 h) [36]. Patients with these factors should be classified as an increased VTE risk group.

### 6. Can a risk-factor stratification based on ethnicity be used to determine proper VTE prophylaxis protocol in Asian patients?

#### Recommendation

Yes, the VTE prophylaxis protocol for Asian patients can be customized by the VTE risk of each ethnicity.

Delegate vote: Agree 86.5%, Disagree 8.1%, Abstain 5.4% (Strong Consensus).

#### Justification

Since the VTE risk after knee and hip arthroplasty or hip fracture surgery in the Asian population is lower than that for Caucasians, the experts agreed that it is justifiable that the VTE prophylaxis protocol can be adjusted from the recommendations for Caucasian patients to suit an Asian patient’s characteristics and economic conditions [7, 28, 35]. A Korean study that analyzed patients undergoing major surgery between 2007 and 2011 found that the overall postoperative VTE rate for major orthopedic procedures was approximately 1.24% [32]. The highest VTE rate was found in hip fracture surgery (approximately 1.60%) [7]. A Taiwanese study analyzed 114,026 patients who underwent hip (*N* = 61,460) or knee (*N* = 52,556) replacement surgery from 2002 to 2006 [37]. This study found an overall incidence of postoperative VTE of 0.44% [37]. The incidence of PE was 4/10,000 for total hip and 7/10,000 for total knee replacement [37].

A meta-analysis showed that the incidence of symptomatic PE was similar in five Asian countries: Taiwan, Japan, Thailand, Korea, and Singapore [35]. When compared with the incidence of DVT in Taiwan, there were no differences in the incidence of overall DVT rate in Japan, but the incidence of overall DVT rate was lower in Thailand, Korea, and Singapore [17]. Other authors identified a higher incidence of proximal DVT in Japan and Thailand but a similar incidence in Korea and Singapore compared to Taiwan’s incidence [25].

A systematic review and meta-analysis showed that the proximal DVT rates were comparable among Asian ethnic groups: 11.8% for Southeast Asians, 11.0% for Japanese, 7.5% for Koreans, and 5.6% for Chinese [7]. The pooled rate was highest in Southeast Asia and lowest in China [20]. There were significant differences between Chinese and Korean patients, between Chinese and Japanese patients, and between Chinese and Southeast Asian patients [29]. Other comparisons were not significantly different [18].

### 7. What is the most useful method or scoring system for stratifying a patient’s risk for VTE before knee and hip arthroplasty or hip fracture surgery?

#### Recommendation

Inconclusive, there is no evidence to prove which method or scoring system provides the best VTE risk stratification.

Delegate vote: Agree 95.9%, Disagree 1.4%, Abstain 2.7% (Strong Consensus).

#### Justification

Ideally, VTE risk assessment and recommended prophylaxis should be specific to the population it serves. Although several studies regarding the method and scoring system for stratifying a patient’s risk for VTE, very few risk assessment models have been validated in the Asian population [38–44].

The Caprini scoring system is a thrombosis risk assessment model that estimates the probability of DVT [38]. Depending on the total risk factor score, patients are grouped into four categories, including low risk, moderate risk, high risk, and highest risk. The Caprini score provides a consistent, accurate, and efficacious method for risk stratification and selection of prophylaxis methods [38]. It has been validated in many versions, such as Spanish, Arabic, and Polish [39]. To date, there have been a few attempts to validate the Caprini risk score of an Asian population [40, 41]. Chinese studies suggest that the Caprini score can effectively stratify hospitalized Chinese patients into VTE risk categories based on individual risk factors [40, 41]. The classification of the highest risk level with a cumulative risk score of ≥ 5 provides distinct clinical information, but further stratification of this group of patients is needed [40, 41]. However, for patients who have undergone TKA, THA, or hip fracture surgery, at least five scores are estimated for this risk assessment model [40, 41]. Therefore, the benefit of this risk assessment model has been brought into question.

Wells scores identify patients as having a low, moderate, or high probability of PE [42]. The reproducibility and reliability of the scores have been validated. However, the original version was criticized as possibly overestimating the overall rate of PE in patients who were considered to have a moderate pretest probability [42]. The Wells score for PE was revised in 2000, to reduce the number of risk categories to two categories: an unlikely group and a likely group [43]. The combination of a score ≤ 4.0 by this simple clinical prediction and a negative D-dimer test can safely rule out PE in a large proportion of patients suspected of being at risk for PE [43]. A study on D-dimers shows that DVT can be ruled out in a patient who is judged clinically unlikely to have DVT combined with a negative D-dimer test [44]. A Doppler ultrasound can then be safely omitted in these patients [44].

Although many methods are available, none were explicitly made for prediction of VTE after knee and hip arthroplasty or hip fracture surgery. The application of such a method should be used in combination with surgical experiences.

### 8. Which patient conditions or factors are associated with elevated VTE risk after knee and hip arthroplasty or hip fracture surgery in an Asian population?

#### Recommendation

Patients conditions or factors that were reported to significantly elevate VTE risk after knee and hip arthroplasty or hip fracture surgery include a history of previous VTE, varicose veins, congestive heart failure, a medical history of thromboembolic stroke, and a family history of VTE.

Delegate vote: Agree 93.2%, Disagree 4.1%, Abstain 2.7% (Strong Consensus).

#### Justification

According to the National Institute for Health and Care Excellence (NICE) guidance of 2010, certain factors have been previously identified to increase the risk or incidence of VTE [45]. These risk factors include (1) active cancer and cancer treatment, (2) age over 60 years, (3) critical care admission, (4) dehydration, (5) thrombophilia, (6) obesity (body mass index [BMI] > 30 kg/m^2^), (7) one or more significant medical comorbidities (e.g., heart disease; metabolic, endocrine or respiratory pathologies; acute infectious diseases; inflammatory conditions), (8) personal history or a first-degree relative with a history of VTE, (9) hormone replacement therapy, (10) use of estrogen-containing contraceptive therapy, (11) varicose veins with phlebitis, and (12) pregnant women or those who have given birth within the previous 6 weeks [45].

##### History of VTE

A meta-analysis showed that the fixed-effect pooled odds ratio (OR) for patients with a history of VTE compared to those without a history of VTE was 11.87 [46]. This finding proved that patients with a history of VTE were at a significant risk of VTE after TKA or THA [46]. An observational cohort study of Asian patients undergoing lower limb major orthopedic surgery also confirmed that a personal history of VTE was a significant independent risk factor for symptomatic VTE, with an OR of 26.9 [29]. Another observational study determined, after adjustment in the multivariate analysis, that a history of VTE was significantly associated with an increased risk of VTE at 3 months after hip surgery [47].

##### Familial history of VTE

Multivariate analysis confirmed that a familial history of VTE was a significant independent risk factor for symptomatic VTE [29].

##### Congestive heart failure

The results of a meta-analysis revealed that congestive heart failure was significantly associated with a higher risk for postoperative VTE (OR, 2.03) [46]. Congestive heart failure was also an independent risk factor for symptomatic VTE, with an OR of 5.1 [29].

##### Varicose veins

A fixed-effect model evaluating 223,249 patients revealed that those with varicose veins were at an elevated risk of VTE (OR, 2.60) [46]. Similarly, a multivariate analysis of the cohort study confirmed that varicose veins were a significant independent risk factor for symptomatic VTE, with an OR of 3.6 [29]. Another cohort study showed that, after adjustment in the multivariate analysis, varicose veins were significantly associated with an increased risk of VTE at 3 months after hip surgery [47].

##### History of stroke

A prospective study demonstrated the result that a patient medical history of thromboembolic stroke was a significant risk factor, which increased the risk of VTE by 4.8 times compared to those without a history of thromboembolic stroke [31].

##### Gender

A case series of an Asian population, after joint arthroplasty without VTE prophylaxis, showed that 3.07% of female patients developed VTE, whereas only 0.32% of male patients developed VTE [31]. This and other findings show that female sex is a significant risk factor for VTE in the Asian population [39]. Similarly, a study that used the Korean Health Insurance Review and Assessment Service database showed that females had a higher relative risk for DVT than males [48]. However, for hip replacement arthroplasty, the relative risk in female patients was similar to that for male patients [48].

##### Old age

Patients aged > 80 were at a higher risk of VTE after TKA or THA [46]. A Korean study showed that, when compared to patients aged < 49 years, the relative risk of DVT was five times higher in patients aged 50–69 and 10 times higher in patients aged > 70 years [48]. However, a prospective study showed the average age of patients with VTE at the time of the occurrence was 79.5 years, which is 12.6 years older than the average age of the patients without VTE, but the difference was not significant [31].

##### Obesity

A prospective study of VTE after joint arthroplasty showed that patients with VTE had an average BMI of 23.03 kg/m^2^, while patients without VTE had a higher average BMI of 25 kg/m^2^ [31]. However, this finding was not statistically significant [40]. Similarly, BMI was not significantly associated with the development of DVT or PE after TKA in patients in an Asian population [49]. By contrast, another prospective study demonstrated that obesity was a significant risk factor of postoperative VTE in an Indian population [50].

##### Active cancer

A meta-analysis showed that the fixed-effect pooled OR for patients with “active” cancer compared to those without “active” cancer was 1.28 [46]. Similarly, malignancy was identified as a significant risk factor in Indian patients [50]. By contrast, a prospective study of hip fracture patients showed that a history of malignancy was not a risk factor [31].

##### Hormonal therapy (HT)

Recent systematic reviews and meta-analyses of the general population show that VTE risk was increased in female oral HT users compared to nonusers, while nonoral HT did not significantly affect VTE risk [31, 51]. However, studies including patients after knee and hip arthroplasty or hip fracture surgery were less founded in the literature [31, 51]. A Canadian study showed no association between postoperative VTE and HT in patients after knee and hip arthroplasty or hip fracture surgery [52].

### 9. In Asian patients, which surgical or perioperative patient management factors are associated with elevated VTE risk after knee and hip arthroplasty or hip fracture surgery?

#### Recommendation

Surgical and perioperative patient management factors that are reported to significantly elevate VTE risk after knee and hip arthroplasty or hip fracture surgery include revision surgery, bilateral surgery, prolonged surgical time, prolonged time to surgery after hip fracture, and delayed ambulation.

Surgical factors and surgical procedures that have an unclear relation to elevated VTE risk after surgery include regional anesthesia (spinal and epidural analgesia), prolonged intraoperative pneumatic tourniquet, and perioperative blood transfusion.

Delegate vote: Agree 95.9%, Disagree 1.4%, Abstain 2.7% (Strong Consensus).

#### Justification

In Asian patients, the significant factors associated with an elevated risk of VTE after knee and hip arthroplasty or hip fracture surgery include revision surgery, bilateral surgery, prolonged surgical time, prolonged time to surgery after hip fracture, and delay ambulation [31, 50]. Some surgical factors and surgical procedures that have an unclear association with an elevated risk of VTE after knee and hip arthroplasty or hip fracture surgery are regional anesthesia, prolonged intraoperative pneumatic tourniquet, and perioperative blood transfusion [31, 50].

##### Bilateral procedure

A prevalence study from Korea suggested that simultaneous bilateral TKA had a significantly higher VTE rate than unilateral arthroplasty [31]. This was due to longer operation time and excessive motion of the joints, which might increase venous stasis and endothelial injury in bilateral arthroplasty [31]. Regarding THA, a meta-analysis showed no difference in the rate of PE between patients with simultaneous bilateral THA and those with staged bilateral THA [53].

##### Revision surgery

Revision surgery is one of the risk factors that elevates VTE risk in patients undergoing hip or knee arthroplasty [31]. Revision surgery presents a 16.6 times higher relative risk of postoperative VTE compared to primary knee and hip arthroplasty [45].

##### Hip fracture surgery

Symptomatic postoperative VTE after hip fracture surgery occurred in 1–9% of patients, according to one study [47]. Prolonged surgical time and prolonged time to surgery after hip fracture are also risk factors of VTE [31]. An operative time of longer than 105 min had a relative risk 1.6 times higher than an operative time of less than 105 min [46].

##### Delayed surgery

Some studies suggested that patients who had a delay of more than two days between the fracture and admission to the hospital had evidence of thrombosis [54, 55]. Patients with a delayed hospital admission had an increased thrombotic incidence [54, 55]. A study in Korean patients found that the preoperative incidence of DVT in hip fractures was relatively low in the Korean geriatric population, but delayed surgery beyond 72 h after injury increased the incidence of DVT [56].

##### Type of anesthesia

A Japanese study showed that spinal anesthesia was significantly associated with an increased risk of VTE in patients undergoing arthroplasty [57]. On the other hand, VTE incidence did not differ between use of general anesthesia and that of combined epidural/general anesthesia [57]. However, surgeons who perform knee or hip arthroplasty for patients who are at high VTE risk may consider avoiding spinal anesthesia, although a careful consideration of the risks and benefits of spinal anesthesia should be conducted [57]. A meta-analysis from five RCTs with 487 THAs also showed no significant difference in occurrence of DVT between general anesthesia and spinal anesthesia [58].

##### Pneumatic tourniquet

An intraoperative pneumatic tourniquet is widely applied in knee arthroplasty [59]. A systematic review of RCTs suggested that prolonged operation time and use of a pneumatic tourniquet increase VTE incidence during knee arthroplasty [59]. However, other studies show that prolonged operation time does not elevate VTE risk after knee and hip arthroplasty [31].

##### Blood transfusions

Blood transfusions can affect the blood clotting cascade, leading to a hypercoagulable state [60, 61]. Perioperative blood transfusion is likely associated with an increased rate of VTE. Some studies show an 8.5% higher incidence of VTE in patients who received a blood transfusion after abdominal surgery [60, 61].

### 10. Which patient factors or conditions are related to bleeding risk following knee and hip arthroplasty or hip fracture surgery in Asian patients?

#### Recommendation

There is no specific study that defined different bleeding risk factors between Asian and other ethnicities.

Delegate vote: Agree 90.5%, Disagree 2.7%, Abstain 6.8% (Strong Consensus).

#### Justification

A multinational cross-sectional study has reported that patient conditions that are considered as increased bleeding risks include advanced age, thrombocytopenia, hemophilia, and other hemorrhagic disorders, including intracranial hemorrhage hepatic impairment, bleeding at hospital admission, active gastroduodenal ulcer, or a known bleeding disorder [62].

ACCP guidelines stated that numerous individual clinical factors had been linked to an increased risk of hemorrhage, including older age, anemia, and renal disease [63]. Furthermore, from an evidence-based review of the literature, the risk of bleeding include age > 65 years, previous bleeding, cancer, metastatic cancer, renal failure, liver failure, thrombocytopenia with platelets < 80,000/mm^3^, previous stroke, diabetes, anemia, antiplatelet therapy, poor anticoagulant control, comorbidity and reduced functional capacity, recent surgery, frequent falls, and alcohol abuse [64].

### 11. Does tranexamic acid (TXA) increase the risk of VTE after knee and hip arthroplasty or hip fracture surgery in Asian patients?

#### Recommendation

No, TXA does not increase VTE risk after knee and hip arthroplasty or hip fracture surgery in Asian patients.

Delegate vote: Agree 95.9%, Disagree 1.4%, Abstain 2.7% (Strong Consensus).

#### Justification

TXA works by inhibiting the activation of plasminogen to prevent fibrin degradation [65]. Because of the properties of antifibrinolytic drugs, there is concern about the increased risk of arterial thromboembolic events and venous thromboembolic events [65]. However, many studies proved that TXA does not increase VTE risk after knee and hip arthroplasty or hip fracture surgery in Asian patients and is safe to use even in patients with increased VTE risk [66–69].

A matched-outcome study showed that patients with a history of VTE had a low risk of recurrent VTE after contemporary TKA and THA, and that rate did not increase with the use of intravenous TXA [65]. Several meta-analyses have shown that TXA is safe to use in patients undergoing knee and hip arthroplasty [66, 67, 70]. Two meta-analyses suggest that TXA can reduce the volume of blood transfusion and transfusion rate. Furthermore, the application of TXA does not increase the risk of DVT or PE [66, 67]. Another meta-analysis demonstrates the lack of evidence of harm from TXA in patients undergoing total joint arthroplasty [70]. Furthermore, moderate evidence supports the safety of TXA in patients undergoing TKA who have an American Society of Anesthesiologists (ASA) score of 3 or higher. The benefits of TXA outweigh the potential risks of thromboembolic events, even in patients with high comorbidities [70]. Current evidences indicate that TXA efficaciously reduces total blood loss and transfusion requirements during knee arthroplasty and hip fracture surgery without significantly increasing the risk of total thromboembolic events, including DVT [68, 69, 71, 72].

Based on the available evidence, it can be concluded that the administration of TXA does not increase VTE risk after knee and hip arthroplasty or hip fracture surgery in Asian patients.

### 12. Are clinical symptoms and signs of DVT (leg pain, leg swelling, distended veins, and skin discoloration) useful for a definite diagnosis of DVT after knee and hip arthroplasty or hip fracture surgery?

#### Recommendation

No, clinical symptoms and signs of DVT should be used in combination with additional investigations for a definite diagnosis of DVT.

Delegate vote: Agree 93.2%, Disagree 2.7%, Abstain 4.1% (Strong Consensus).

#### Justification

All patients undergoing knee and hip arthroplasty or hip fracture surgery have an inherent risk of venous thromboembolic complications [73, 74]. Since leg pain, leg swelling, and discoloration are common and nonspecific after knee and hip surgery, they should not be solely used for diagnosis of DVT after knee and hip arthroplasty or hip fracture surgery [7, 73]. These signs and symptoms can also confuse DVT and other medical conditions such as heart failure or local infection [75–77].

During the first 4 weeks after major surgery, a localized tenderness along the distribution of the deep venous system, entire leg swelling, calf swelling by more than 3 cm when compared with the opposite leg, pitting edema, or the presence of collateral superficial veins are highly suspicious signs of DVT [78]. Further investigations should be performed for a definite diagnosis [78]. However, the absence of clinical symptoms and signs of DVT can help reduce the number of diagnostic tests required for patients after knee and hip arthroplasty or hip fracture surgery [78, 79].

Therefore, these clinical symptoms and signs are not reliable for the diagnosis of DVT in patients after knee and hip arthroplasty or hip fracture surgery. They should alert orthopedic surgeons to consider additional investigations for a definite diagnosis.

### 13. Can PE occur without clinical symptoms and signs of DVT?

#### Recommendation

Yes, a PE can occur without clinical symptoms and signs of DVT.

Delegate vote: Agree 100%, Disagree 0%, Abstain 0% (Unanimous Consensus).

#### Justification

It is believed that the most common source of PE is untreated or undetected DVT, primarily from the pelvis and lower extremities [80]. However, most patients with PE do not have detectable DVT. Only 20% of the patients with PE have an identifiable DVT [80]. When PE occurs without clinical symptoms and signs of DVT, the condition is termed “de novo PE” [81, 82]. Regarding PE in trauma or postoperative hip fracture surgery, it has been proposed that the clot may not originate from deep veins but may occur de novo in the lungs because of endothelial inflammatory response [83].

### 14A. Is routine postoperative screening for DVT necessary in knee and hip arthroplasty?

#### Recommendation

No, routine screening for DVT after knee and hip arthroplasty is not necessary.

Delegate vote: Agree 94.6%, Disagree 2.7%, Abstain 2.7% (Strong Consensus).

### 14B. Is routine postoperative screening for DVT necessary in hip fracture surgery?3.15.1. Recommendation

Inconclusive; there is no evidence to support routine postoperative screening for DVT.

Delegate vote: Agree 94.6%, Disagree 4.1%, Abstain 1.3% (Strong Consensus).

#### Justification

VTE can occur up to 3 months after TKA and THA and is also a common cause of readmission after THA [84, 85]. Routine screening after knee and hip arthroplasty cannot completely rule out postoperative DVT [84, 85]. In THA patients with negative venography at discharge, 20% will develop new DVT within 3 months without VTE prophylaxis [84–86]. If VTE prophylaxis is given, the rates of symptomatic VTE will be reduced to 1.3–10% [84–86]. AAOS and ACCP clinical practice guidelines are in agreement and suggest against the routine use of ultrasound for the screening of DVT in patients after knee or hip arthroplasty [3, 5].

Patients undergoing hip fracture surgery have a higher risk of DVT and PE [3]. Regardless of these findings, ACCP guidelines do not recommend routine ultrasound screening before hospital discharge for asymptomatic patients after hip fracture surgery [3]. However, a recent study proposed routine screening for DVT before surgery because of a high incidence of VTE in femoral neck fractures [87].

### 15. Is duplex ultrasonography a preferred initial investigation for diagnosis of DVT after knee and hip arthroplasty or hip fracture surgery?

#### Recommendation

Yes, duplex ultrasonography is a useful initial investigation for the diagnosis of DVT after knee and hip arthroplasty or hip fracture surgery.

Delegate vote: Agree 97.2%, Disagree 1.4%, Abstain 1.4% (Strong Consensus).

#### Justification

Duplex ultrasound is a noninvasive diagnostic tool for DVT and has now largely replaced contrast venography as the preferred test for diagnosing clinically suspected DVT [77, 88–90]. For the diagnosis of proximal DVT, duplex ultrasonography provides a sensitivity of 94–97% and a specificity of 98% [77, 89, 91]. Moreover, ultrasonography has a positive predictive value of 100% and a negative predictive value of 100% for symptomatic DVT. However, the positive predictive value and negative predictive value for asymptomatic DVT are 71% and 94%, respectively [77, 89, 91]. The duplex ultrasound sensitivity for the diagnosis of distal DVT is relatively low at 57%, and it is only 48% for detecting asymptomatic calf vein thrombosis [77].

NICE has approved the recommendations for the request of proximal leg vein ultrasound scan in patients suspected of DVT with a Wells score ≥ 2 (“DVT likely”) [92]. In patients with a Wells score < 1 (“DVT unlikely”), ultrasound should be done if the D-dimer test is positive [92].

### 16. In controversial cases, what is the investigation for a definitive diagnosis of DVT?

#### Recommendation

Lower leg venography, including conventional contrast venography, computed tomography (CT) venography, and magnetic resonance venography, can be used for a definitive diagnosis of DVT in controversial cases.

Delegate vote: Agree 93.2%, Disagree 5.4%, Abstain 1.4% (Strong Consensus).

#### Justification

Leg swelling and pain can be suspicious signs of postoperative DVT [73, 74]. Special investigations may be required for proper evaluation [78, 79]. However, there is no single ideal investigation for the diagnosis of DVT [78, 79].

Conventional contrast venography has been historically quoted as a gold standard for diagnosing DVT, with a reported sensitivity of 70–100% and a specificity of 60–88% [77, 88]. However, conventional contrast venography is invasive, not readily available, and contraindicated in patients with renal insufficiency or allergy to the contrast medium [77].

Recently, newer imaging technology, such as CT venography and magnetic resonance venography, have been proposed as alternatives to conventional contrast venography and duplex ultrasound, with comparable sensitivity and specificity [77]. Moreover, nonimaging methods, such as standardized clinical probability assessment, and laboratory tests, such as a D-dimer test, can be safely used to rule out acute DVT [93].

Therefore, conventional contrast venography is now rarely necessary for DVT diagnosis [94]. However, it remains as a referential standard for clinical research and can be utilized when results from other methods are inconclusive [94].

### 17. What is the gold standard investigation for the diagnosis of PE?

#### Recommendation

The current gold standard diagnostic investigation is pulmonary angiography. However, a computed tomographic pulmonary angiogram (CTPA) is the more preferred investigation for PE diagnosis.

Delegate vote: Agree 98.6%, Disagree 1.4%, Abstain 0% (Strong Consensus).

#### Justification

PE is a potentially fatal complication following knee and hip arthroplasty and hip fracture surgery [88]. Clinical symptoms and signs alone have limited use for the diagnosis of PE [88]. Currently, pulmonary angiography is the gold standard diagnostic tool for PE [88, 95, 96]. However, CTPA may be an alternative investigation for the diagnosis of PE [88, 95, 96]. CTPA is the recommended imaging modality for investigation of acute PE, with a diagnostic sensitivity of 57–100% and specificity of 78–100% [95]. However, it should be undertaken after an assessment of the probability of PE. A recent study has shown that a combination of risk assessment, D-dimer testing, and CTPA is the most preferred diagnostic method for diagnosing PE [97, 98]. Ventilation-perfusion scanning is an alternative diagnostic tool for PE in patients who are contraindicated for intravenous contrast media, e.g., patients who have renal failure or contrast material allergies or young females and patients who cannot fit into the CT scanner [99].

## Data Availability

Not applicable.

## Abbreviations

ACCP: American College of Chest Physicians
AAOS: American Academy of Orthopaedic Surgeons
AP: Asia-Pacific
BMI: Body mass index
CTPA: Computed tomographic pulmonary angiogram
DVT: Deep vein thrombosis
HT: Hormonal therapy
PE: Pulmonary embolism
PFA: Patellofemoral arthroplasty
RCT: Randomized controlled trial
THA: Total hip arthroplasty
TJA: Total joint arthroplasty
TKA: Total knee arthroplasty
TXA: Tranexamic acid
UKA: Unicompartmental knee arthroplasty
VTE: Venous thromboembolism

## References

[1] van Oosterom N, Barras M, Bird R, Nusem I, Cottrell N (2020). A narrative review of aspirin resistance in VTE prophylaxis for orthopaedic surgery. Drugs..

[2] Wilson DG, Poole WE, Chauhan SK, Rogers BA (2016). Systematic review of aspirin for thromboprophylaxis in modern elective total hip and knee arthroplasty. Bone Joint J.

[3] Falck-Ytter Y, Francis CW, Johanson NA, Curley C, Dahl OE, Schulman S, Ortel TL, Pauker SG, Colwell CW (2012). Prevention of VTE in orthopedic surgery patients: Antithrombotic Therapy and Prevention of Thrombosis, 9th ed: American College of Chest Physicians Evidence-Based Clinical Practice Guidelines. Chest..

[4] Mont MA, Jacobs JJ (2011). AAOS clinical practice guideline: preventing venous thromboembolic disease in patients undergoing elective hip and knee arthroplasty. J Am Acad Orthop Surg..

[5] Mont MA, Jacobs JJ, Boggio LN, Bozic KJ, Della Valle CJ, Goodman SB, Lewis CG, Yates AJ, Jr, Watters WC, 3rd, Turkelson CM, Wies JL, Donnelly P, Patel N, Sluka P. Preventing venous thromboembolic disease in patients undergoing elective hip and knee arthroplasty. J Am Acad Orthop Surg 2011;19:768–776, 12, doi: 10.5435/00124635-201112000-00007 10.5435/00124635-201112000-00007 22134209

[6] Cote MP, Chen A, Jiang Y, Cheng V, Lieberman JR (2017). Persistent pulmonary embolism rates following total knee arthroplasty even with prophylactic anticoagulants. J Arthroplast.

[7] Kanchanabat B, Stapanavatr W, Meknavin S, Soorapanth C, Sumanasrethakul C, Kanchanasuttirak P (2011). Systematic review and meta-analysis on the rate of postoperative venous thromboembolism in orthopaedic surgery in Asian patients without thromboprophylaxis. Br J Surg.

[8] Diamond IR, Grant RC, Feldman BM, Pencharz PB, Ling SC, Moore AM, Wales PW (2014). Defining consensus: a systematic review recommends methodologic criteria for reporting of Delphi studies. J Clin Epidemiol.

[9] Custer RL, Scarcella JA, Stewart BR (1999). The modified Delphi technique-A rotational modification. J Career Tech Educ.

[10] Aengenheyster S, Cuhls K, Gerhold L, Heiskanen-Schüttler M, Huck J, Muszynska M (2017). Real-time Delphi in practice—a comparative analysis of existing software-based tools. Technol Forecast Soc Change.

[11] Joshi A, Kale S, Chandel S, Pal DK (2015) Likert Scale: Explored and Explained. Curr J Appl Sci Technol 7(4):396-403. 10.9734/BJAST/2015/14975.

[12] Scuderi GR, Scott WN, Tchejeyan GH (2001). The Insall legacy in total knee arthroplasty. Clin Orthop Relat Res.

[13] Murray DW, Parkinson RW (2018). Usage of unicompartmental knee arthroplasty. Bone Joint J.

[14] Pisanu G, Rosso F, Bertolo C, Dettoni F, Blonna D, Bonasia DE, Rossi R (2017). Patellofemoral arthroplasty: current concepts and review of the literature. Joints..

[15] Thomas BJ, Saa J, Lane JM (1996). Total hip arthroplasty. Curr Opin Rheumatol.

[16] Pivec R, Johnson AJ, Mears SC, Mont MA (2012). Hip arthroplasty. Lancet..

[17] Seyler TM, Cui Q, Mihalko WM, Mont MA, Saleh KJ (2007). Advances in hip arthroplasty in the treatment of osteonecrosis. Instr Course Lect.

[18] Bhattacharyya T, Koval KJ (2009). Unipolar versus bipolar hemiarthroplasty for femoral neck fractures: is there a difference?. J Orthop Trauma.

[19] van Gerwen M, Shaerf DA, Veen RM (2010). Hip resurfacing arthroplasty. Acta Orthop.

[20] Veronese N, Maggi S (2018). Epidemiology and social costs of hip fracture. Injury..

[21] Griffin T, Rowden N, Morgan D, Atkinson R, Woodruff P, Maddern GJ (2007). Unicompartmental knee arthroplasty for the treatment of unicompartmental osteoarthritis: a systematic study. ANZ J Surg.

[22] Wilson HA, Middleton R, Abram SGF, Smith S, Alvand A, Jackson WF, Bottomley N, Hopewell S, Price AJ (2019). Patient relevant outcomes of unicompartmental versus total knee replacement: systematic review and meta-analysis. BMJ..

[23] Yang K, Wang M, Yeo S, Lo NN (2003). Minimally invasive unicondylar versus total condylar knee arthroplasty—early results of a matched-pair comparison. Singapore Med J.

[24] Willis-Owen C, Sarraf K, Martin A, Martin D (2011). Are current thrombo-embolic prophylaxis guidelines applicable to unicompartmental knee replacement?. J Bone Joint Surg Br.

[25] Liodakis E, Antoniou J, Zukor DJ, Huk OL, Epure LM, Bergeron SG (2016). Major complications and transfusion rates after hemiarthroplasty and total hip arthroplasty for femoral neck fractures. J Arthroplasty.

[26] Ravi B, Pincus D, Khan H, Wasserstein D, Jenkinson R, Kreder HJ (2019). Comparing complications and costs of total hip arthroplasty and hemiarthroplasty for femoral neck fractures: a propensity score-matched, population-based study. J Bone Joint Surg Am.

[27] Yoon RS, Mahure SA, Hutzler LH, Iorio R, Bosco JA (2017). Hip arthroplasty for fracture vs elective care: one bundle does not fit all. J Arthroplasty.

[28] Abd Razak HRB, Abd Razak NFB, H-CA T (2017). J Arthroplasty.

[29] Leizorovicz A, Turpie A, Cohen A, Wong L, Yoo M, Dans A (2005). Epidemiology of venous thromboembolism in Asian patients undergoing major orthopedic surgery without thromboprophylaxis. The SMART Study. J Thrombosis Haemostasis.

[30] Okike K, Chan PH, Prentice HA, Navarro RA, Hinman AD, Paxton EW (2019). Association of race and ethnicity with total hip arthroplasty outcomes in a universally insured population. J Bone Joint Surg Am.

[31] Won M-H, Lee G-W, Lee T-J, Moon K-H (2011). Prevalence and risk factors of thromboembolism after joint arthroplasty without chemical thromboprophylaxis in an Asian population. J Arthroplasty.

[32] Yhim HY, Jang MJ, Bang SM, Kim K, Kim YK, Nam SH, Bae S, Kim SH, Mun YC, Kim I (2014). Incidence of venous thromboembolism following major surgery in Korea: from the Health Insurance Review and Assessment Service database. J Thromb Haemost.

[33] Kim K-I, Cho K-Y, Jin W, Khurana SS, Bae DK (2011). Recent Korean perspective of deep vein thrombosis after total knee arthroplasty. J Arthroplasty.

[34] Geerts WH, Pineo GF, Heit JA, Bergqvist D, Lassen MR, Colwell CW, Ray JG (2004). Prevention of venous thromboembolism: the Seventh ACCP Conference on Antithrombotic and Thrombolytic Therapy. Chest.

[35] Lee WS, Kim KI, Lee HJ, Kyung HS, Seo SS (2013). The incidence of pulmonary embolism and deep vein thrombosis after knee arthroplasty in Asians remains low: a meta-analysis. Clin Orthop Relat Res.

[36] Z-h Z, Shen B, Yang J, Zhou Z-K, Pei F-X (2015). Risk factors for venous thromboembolism of total hip arthroplasty and total knee arthroplasty: a systematic review of evidences in ten years. BMC Musculoskelet Disord.

[37] Wu PK, Chen CF, Chung LH, Liu CL, Chen WM (2014). Population-based epidemiology of postoperative venous thromboembolism in Taiwanese patients receiving hip or knee arthroplasty without pharmacological thromboprophylaxis. Thromb Res.

[38] Caprini JA (2005). Thrombosis risk assessment as a guide to quality patient care. Dis Mon.

[39] Paz Rios LH, Fuentes HE, Oramas DM, Andrade XA, Al-Ogaili A, Iskander M, Iskander F, Iskandar ANA, Kowacz W, Iwanski A (2018). Validation of a patient-completed Caprini risk assessment tool for Spanish, Arabic, and Polish speakers. Clin Appl Thromb Hem.

[40] Zhou H, Wang L, Wu X, Tang Y, Yang J, Wang B, Yan Y, Liang B, Wang K, Ou X (2014). Validation of a venous thromboembolism risk assessment model in hospitalized Chinese patients: a case-control study. Atheroscler Thromb.

[41] Zhou H-X, Peng L-Q, Yan Y, Yi Q, Tang Y-J, Shen Y-C, Feng Y-L, Wen F-Q (2012). Validation of the Caprini risk assessment model in Chinese hospitalized patients with venous thromboembolism. Thromb Res.

[42] Wells PS, Ginsberg JS, Anderson DR, Kearon C, Gent M, Turpie AG, Bormanis J, Weitz J, Chamberlain M, Bowie D (1998). Use of a clinical model for safe management of patients with suspected pulmonary embolism. Ann Intern Med.

[43] Wells PS, Anderson DR, Rodger M, Ginsberg JS, Kearon C, Gent M, Turpie AG, Bormanis J, Weitz J, Chamberlain M (2000). Derivation of a simple clinical model to categorize patients probability of pulmonary embolism: increasing the models utility with the SimpliRED D-dimer. Thromb Haemost.

[44] Wells PS, Anderson DR, Rodger M, Forgie M, Kearon C, Dreyer J, Kovacs G, Mitchell M, Lewandowski B, Kovacs MJ (2003). Evaluation of D-dimer in the diagnosis of suspected deep-vein thrombosis. N Engl J Med.

[45] Hill J, Treasure T (2010). Reducing the risk of venous thromboembolism (deep vein thrombosis and pulmonary embolism) in patients admitted to hospital: summary of the NICE guideline. Heart.

[46] Zhang J, Chen Z, Zheng J, Breusch SJ, Tian J (2015). Risk factors for venous thromboembolism after total hip and total knee arthroplasty: a meta-analysis. Arch Orthop Trauma Surg.

[47] Rosencher N, Vielpeau C, Emmerich J, Fagnani F, Samama CM (2005). Venous thromboembolism and mortality after hip fracture surgery: the ESCORTE study. Thromb Haemost.

[48] Lee SY, Ro DH, Chung CY, Lee KM, Kwon S-S, Sung KH, Park MS (2015). Incidence of deep vein thrombosis after major lower limb orthopedic surgery: analysis of a nationwide claim registry. Yonsei Med J.

[49] Tay K, Abd Razak HRB, Tan AH (2016). Obesity and venous thromboembolism in total knee arthroplasty patients in an Asian population. J Arthroplasty.

[50] Bagaria V, Modi N, Panghate A, Vaidya S (2006). Incidence and risk factors for development of venous thromboembolism in Indian patients undergoing major orthopaedic surgery: results of a prospective study. Postgrad Med J.

[51] Rovinski D, Ramos RB, Fighera TM, Casanova GK, Spritzer PM (2018). Risk of venous thromboembolism events in postmenopausal women using oral versus non-oral hormone therapy: A systematic review and meta-analysis. Thrombosis Res.

[52] Schiff RL, Kahn SR, Shrier I, Strulovitch C, Hammouda W, Cohen E, Zukor D (2005). Identifying orthopedic patients at high risk for venous thromboembolism despite thromboprophylaxis. Chest.

[53] Shao H, Chen C-L, Maltenfort MG, Restrepo C, Rothman RH, Chen AF (2017). Bilateral total hip arthroplasty: 1-stage or 2-stage? A meta-analysis. J Arthroplasty.

[54] Hefley WF, Nelson CL, Puskarich-May CL (1996). Effect of delayed admission to the hospital on the preoperative prevalence of deep-vein thrombosis associated with fractures about the hip. J Bone Joint Surg Am.

[55] Zahn HR, Skinner JA, Porteous MJ (1999). The preoperative prevalence of deep vein thrombosis in patients with femoral neck fractures and delayed operation. Injury.

[56] Cho Y-H, Byun Y-S, Jeong D-G, Han I-H, Park Y-B (2015). Preoperative incidence of deep vein thrombosis after hip fractures in Korean. Clin Orthop Surg.

[57] Nakamura M, Kamei M, Bito S, Migita K, Miyata S, Kumagai K, Abe I, Nakagawa Y, Nakayama Y, Saito M (2017). Spinal anesthesia increases the risk of venous thromboembolism in total arthroplasty: secondary analysis of a J-PSVT cohort study on anesthesia. Medicine.

[58] Pu X, Sun J-M (2019). General anesthesia vs spinal anesthesia for patients undergoing total-hip arthroplasty: a meta-analysis. Medicine (Baltimore).

[59] Xing KH, Morrison G, Lim W, Douketis J, Odueyungbo A, Crowther M (2008). Has the incidence of deep vein thrombosis in patients undergoing total hip/knee arthroplasty changed over time? A systematic review of randomized controlled trials. Thromb Res.

[60] Abu-Rustum NR, Richard S, Wilton A, Lev G, Sonoda Y, Hensley ML, Gemignani M, Barakat RR, Chi DS (2005). Transfusion utilization during adnexal or peritoneal cancer surgery: effects on symptomatic venous thromboembolism and survival. Gynecol Oncol.

[61] Helm JH, Helm MC, Kindel TL, Gould JC, Higgins RM (2019). Blood transfusions increase the risk of venous thromboembolism following ventral hernia repair. Hernia..

[62] Anderson FA, Spencer FA (2003). Risk factors for venous thromboembolism. Circulation.

[63] Cohen AT, Tapson VF, Bergmann J-F, Goldhaber SZ, Kakkar AK, Deslandes B, Huang W, Zayaruzny M, Emery L, Anderson FA (2008). Venous thromboembolism risk and prophylaxis in the acute hospital care setting (ENDORSE study): a multinational cross-sectional study. Lancet.

[64] Kearon C, Akl EA, Comerota AJ, Prandoni P, Bounameaux H, Goldhaber SZ, Nelson ME, Wells PS, Gould MK, Dentali F (2012). Antithrombotic therapy for VTE disease: antithrombotic therapy and prevention of thrombosis: American College of Chest Physicians evidence-based clinical practice guidelines. Chest.

[65] Sabbag OD, Abdel MP, Amundson AW, Larson DR, Pagnano MW (2017). Tranexamic acid was safe in arthroplasty patients with a history of venous thromboembolism: a matched outcome study. J Arthroplasty.

[66] Wei Z, Liu M (2015). The effectiveness and safety of tranexamic acid in total hip or knee arthroplasty: a meta-analysis of 2720 cases. Transfus Med.

[67] Yang Z-G, Chen W-P, Wu L-D (2012). Effectiveness and safety of tranexamic acid in reducing blood loss in total knee arthroplasty: a meta-analysis. J Bone Joint Surg Am.

[68] Xiao C, Zhang S, Long N, Yu W, Jiang Y (2019). Is intravenous tranexamic acid effective and safe during hip fracture surgery? An updated meta-analysis of randomized controlled trials. Arch Orthop Trauma Surg.

[69] Lee SH, Cho K-Y, Khurana S, Kim K-I (2013). Less blood loss under concomitant administration of tranexamic acid and indirect factor Xa inhibitor following total knee arthroplasty: a prospective randomized controlled trial. Knee Surg Sports Traumatol Arthrosc.

[70] Fillingham YA, Ramkumar DB, Jevsevar DS, Yates AJ, Shores P, Mullen K, Bini SA, Clarke HD, Schemitsch E, Johnson RL (2018). The safety of tranexamic acid in total joint arthroplasty: a direct meta-analysis. J Arthroplasty.

[71] Farrow LS, TO S, Ashcroft GP, Myint PK (2016). A systematic review of tranexamic acid in hip fracture surgery. Br J Clin Pharmacol.

[72] Y-m Q, Wang H-p, Y-j L, Ma B-b, Xie T, Wang C, Chen H, Rui Y-F (2019). The efficacy and safety of intravenous tranexamic acid in hip fracture surgery: a systematic review and meta-analysis. J Orthop Trans.

[73] Anand SS, Wells PS, Hunt D, Brill-Edwards P, Cook D, Ginsberg JS (1998). Does this patient have deep vein thrombosis?. JAMA.

[74] Bawa H, Weick JW, Dirschl DR, Luu HH (2018). Trends in deep vein thrombosis prophylaxis and deep vein thrombosis rates after total hip and knee arthroplasty. J Am Acad Orthop Surg.

[75] Elias A, Mallard L, Elias M, Alquier C, Guidolin F, Gauthier B, Viard A, Mahouin P, Vinel A, Boccalon H (2003). A single complete ultrasound investigation of the venous network for the diagnostic management of patients with a clinically suspected first episode of deep venous thrombosis of the lower limbs. Thromb Haemost.

[76] Grüne S, Orlik J, Von Korn H, Schacherer D, Schlottmann K, Brünnler T (2011). Clinical signs in the diagnosis of deep vein thrombosis. Int Angiol.

[77] Wang K-L, Chu P-H, Lee C-H, Pai P-Y, Lin P-Y, Shyu K-G, Chang W-T, Chiu K-M, Huang C-L, Lee C-Y (2016). Management of venous thromboembolisms: part I The consensus for deep vein thrombosis. Acta Cardiol Sin.

[78] Wells PS, Anderson DR, Bormanis J, Guy F, Mitchell M, Gray L, Clement C, Robinson KS, Lewandowski B (1997). Value of assessment of pretest probability of deep-vein thrombosis in clinical management. Lancet.

[79] Tovey C, Wyatt S (2003). Diagnosis, investigation, and management of deep vein thrombosis. BMJ.

[80] Knudson MM, Gomez D, Haas B, Cohen MJ, Nathens AB (2011). Three thousand seven hundred thirty-eight posttraumatic pulmonary emboli: a new look at an old disease. Ann Surg.

[81] Kahn SA, Schubmehl H, Stassen NA, Sangosanya A, Cheng JD, Gestring ML, Bankey PE (2013). Risk factors associated with venous thromboembolism in isolated blunt chest trauma. Am Surg.

[82] Schwartz T, Hingorani A, Ascher E, Marks N, Shiferson A, Jung D, Jimenez R, Jacob T (2012). Pulmonary embolism without deep venous thrombosis. Ann Vasc Surg.

[83] Velmahos GC, Spaniolas K, Tabbara M, Abujudeh HH, de Moya M, Gervasini A, Alam HB (2009). Pulmonary Embolism and Deep Venous Thrombosis in Trauma: Are They Related?. Arch Surg.

[84] Geerts WH, Bergqvist D, Pineo GF, Heit JA, Samama CM, Lassen MR, Colwell CW (2008). Prevention of venous thromboembolism: American College of Chest Physicians Evidence-Based Clinical Practice Guidelines (8th Edition). Chest.

[85] White RH, Romano PS, Zhou H, Rodrigo J, Bargar W (1998). Incidence and time course of thromboembolic outcomes following total hip or knee arthroplasty. Arch Intern Med.

[86] Flevas DA, Megaloikonomos PD, Dimopoulos L, Mitsiokapa E, Koulouvaris P, Mavrogenis AF (2018). Thromboembolism prophylaxis in orthopaedics: an update. EFORT Open Rev.

[87] Xia ZN, Xiao K, Zhu W, Feng B, Zhang BZ, Lin J, Qian WW, Jin J, Gao N, Qiu GX, Weng XS (2018). Risk assessment and management of preoperative venous thromboembolism following femoral neck fracture. J Orthop Surg Res.

[88] Saleh J, El-Othmani MM, Saleh KJ (2017). Deep vein thrombosis and pulmonary embolism considerations in orthopedic surgery. Orthop Clin North Am.

[89] Segal JB, Eng J, Tamariz LJ, Bass EB (2007). Review of the evidence on diagnosis of deep venous thrombosis and pulmonary embolism. Ann Fam Med.

[90] Swanson E (2015). Ultrasound screening for deep venous thrombosis detection: a prospective evaluation of 200 plastic surgery outpatients. Plast Reconstr Surg Glob Open.

[91] Kearon C, Julian JA, Newman TE, Ginsberg JS (1998). Noninvasive diagnosis of deep venous thrombosis. McMaster Diagnostic Imaging Practice Guidelines Initiative. Ann Intern Med.

[92] Chong LY, Fenu E, Stansby G, Hodgkinson S (2012). Management of venous thromboembolic diseases and the role of thrombophilia testing: summary of NICE guidance. BMJ..

[93] Righini M, Perrier A, De Moerloose P, Bounameaux H (2008). D-Dimer for venous thromboembolism diagnosis: 20 years later. J Thromb Haemost.

[94] Bates SM, Jaeschke R, Stevens SM, Goodacre S, Wells PS, Stevenson MD, Kearon C, Schunemann HJ, Crowther M, Pauker SG, Makdissi R, Guyatt GH (2012). Diagnosis of DVT: Antithrombotic Therapy and Prevention of Thrombosis, 9th ed: American College of Chest Physicians Evidence-Based Clinical Practice Guidelines. Chest..

[95] Alhassan S, Leap J, Popuri A, Yadam S, Singh AC, Balaan M (2017). Diagnostic considerations of venous thromboembolic disease. Crit Care Nurs Q.

[96] Stein PD, Athanasoulis C, Alavi A, Greenspan RH, Hales CA, Saltzman HA, Vreim CE, Terrin ML, Weg JG (1992). Complications and validity of pulmonary angiography in acute pulmonary embolism. Circulation..

[97] (2003) British Thoracic Society. British Thoracic Society guidelines for the management of suspected acute pulmonary embolism. Thorax. 58(6):470–483. 10.1136/thorax.58.6.470 10.1136/thorax.58.6.470 PMC1746692 12775856

[98] Gao H, Liu H, Li Y (2018). Value of D-dimer levels for the diagnosis of pulmonary embolism: an analysis of 32 cases with computed tomography pulmonary angiography. Exp Ther Med.

[99] Moore AJE, Wachsmann J, Chamarthy MR, Panjikaran L, Tanabe Y, Rajiah P (2018). Imaging of acute pulmonary embolism: an update. Cardiovasc Diagn Ther.

